# Cases of Cerebro-Spinal Meningitis in Cook County Hospital

**Published:** 1872-04-15

**Authors:** E. F. Ingals

**Affiliations:** House Physician


### CASES OF CEREBRO-SPINAL MENINGITIS IN COOK COUNTY HOSPITAL.

REPORTED BY E. F. INGALS, M.D., HOUSE PHYSICIAN.

*Case* 1.—H—— O——, æt. 40; harness-maker; German. Admitted March 9th, 1872.

For one week before admission the patient had been complaining of severe headache, much thirst and loss of appetite, with some pain in the middle of the back.

On admission the patient was mildly delirious, especially at night. He still complained of headache, pain in the lumbar regions, thirst and anorexia. His hearing was poor, the pupils contracted, face congested, and tongue slightly coated. The patient had a mania for injections, sometimes getting up in the night, stealing quietly into the bath-room, and proceeding to take an enema. On one occasion he fainted in attempting to take the enema, and was found by the nurse, wholly unconscious.

Constant care was necessary to prevent him from making these experiments, even after his bowels had been freely moved by a cathartic.

After a few days the right pupil became dilated, the pain in the head and back began to subside, and the delirium moderated. Still the patient slept poorly; he had no appetite, and there was a constant tendency to constipation. About this time double vision began to be complained of, and dizziness on attempting to sit up.

The more violent symptoms gradually subsided until, in the course of a couple of weeks after admission, the patient was able to be up for a short time; his cephalalgia was not continuous; the appetite was improved; there was no great amount of thirst, and no delirium; however, after being up for a short time the patient would become dizzy and the headache would recur.

All the symptoms in this case have gradually subsided, and at present the right pupil is only slightly dilated, there is a good appetite, the bowels are easily regulated, and the patient is able to be up for three or four hours at a time. The hearing seems somewhat improved, but is still poor.

*Case* 2.—The second case is that of a man who was admitted to the hospital suffering from severe pain in the head, neck, and lumbar region. We were unable to get a perfect history of this case. On coming in, he stated that he had been sick ten days; that the attack commenced with a chill, and that he was continually growing worse.

On admission, the face was flushed, brows knit, tongue coated white, pulse moderately accelerated, appetite poor, and bowels constipated. No symptoms of abdominal or thoracic diseases could be elicited. The question arose as to whether this was not rheumatism; but the want of rheumatic symptoms in other parts, and the persistence of the disease, together with the epidemic influences prevailing, led us to suppose it a case of mild cerebro-spinal meningitis. In this case free catharsis was induced, and 15 or 20 gr. doses of potass. brom. were given every three or four hours, for a couple of days.

Subsequently tinct. cannabis ind. was employed in doses at first of ♏xx, but rapidly increased to ʒ i, and given every two or three hours, while there was pain in the head. The patient experienced much relief from the latter drug. He slowly progressed toward recovery. As the more active symptoms subsided, potass. iod. administered and blisters applied behind ears. 28 days after his sickness commenced he was so far recovered as to leave the hospital.

*Case* 3.—H. G——, 20; England; mason. Admitted March 11th.

The evening of admission the patient was more rational. He stated that ten days previously he had been attacked with fever and severe pain in the head and neck. He complained of loss of appetite, thirst, severe pains in head, neck and back, sleeplessness and constipation. He stated that the bowels had moved but once in ten days.

The patient lay with his head drawn back; and he was unable to bend it forward, pressure over the cervical and lumbar portions of the spine greatly aggravated the pain in these parts. The tongue was coated white, and moist excepting at the tip; pulse 100; temperature 101½°. A free cathartic was ordered (hydrarg chlor. mit. et rhei. aa gr. x), to be followed by potass. brom. gr. x, every three hours. March 13, prescribed opium gr. i, and ext. hyos. gr. 1-4, at bed-time. Herpes labialis appeared at this time. March 14. Last night patient slept well for the first time since he was taken sick; can move the head some this morning. Subsequently to these entries the patient gradually improved on the use of tr. cannabis ind. with opium and hyos. at night. After the active symptoms had passed potass. iod. was given. 28 days after admission, and 38 after the beginning of the disease, the patient was so far recovered as to leave the hospital.

*Case* 4.—M. J. S——, 33; servant; Canada. Admitted March 6th.

Patient was brought into the hospital in a condition of wild delirium; head drawn back and held firmly. In the evening, chlor. hyd. ʒ i was ordered given in doses of gr. xv, every half hour. It gave very little relief.

March 7. Ordered ʒ ii of chlo. hyd. in divided doses at evening. Patient very delirious, requiring to be tied in bed. When loosened, she would throw herself from bed and resist forcibly all efforts to return her; pulse moderately accelerated, and face flushed.

March 8. The chloral administered last evening seemed only to increase the delirium. Thinking that constipation might have existed for some time, we ordered ol. tiglii; to relieve the delirium and evident pain in the head and back of neck, potass. brom. ℈i, every three hours was administered. 2 p. m. Pulse about 100; face flushed; temperature 104¼°. Delirium continues. 7.30 p.m. Temperature 102°; patient still noisy and delirious; had fever during forenoon.

March 9. Temperature 100°; patient more quiet; she slept three or four hours during the night; considerable fever occurred in the forenoon; the bowels responded actively to the croton oil.

March 10. The patient did not sleep last night, and is very restless this morning. The nurse says that the fever was much earlier to-day. The face is flushed; tongue dry and fissured, though not much coated; bowels soluble. Brom. potass. continued.

March 13. Pulse 112; temperature 102¼°; tongue dry and slightly coated; the patient rested better last night than she has since coming into the house. Yesterday there were two evacuations of hardened fæces.

Ordered a cathartic. We have as yet been unable to obtain any history of the patient previous to her admission, except that she was found by an officer sick in a street-car, and that he brought her to the hospital.

March 17. Patient has been gradually improving since the last entry, and has been able to complain of the pain in her head. Last night tinct. cannabis ind. was ordered, and gave much relief to the pain. This morning she is very much improved, and is able for the first time to give her name. She says that she had been complaining of pain in the head, neck, and back, for a week before coming to the hospital. She has an indistinct recollection of being brought here. Subsequently to this time the patient continued gradually to improve. Headache frequently recurred, which was treated with tinct. cannabis ind. Potass. iod. was also administered continuously for a couple of weeks.

At present the patient is nearly well. She is able to be up considerable of the time, but is occasionally affected with dizziness and headache. Her hearing has been impaired by the disease, and her mind seems to operate sluggishly, but whether this latter is an effect of the disease or not cannot be accurately determined, as we have none of her previous history.

*Case* 5.—W—— B.; 17; laborer; England. Admitted April 1st.

Patient stated on admission that he had been sick about four days. While at work he was attacked with severe pain in the temples and back of the neck. Before coming to the hospital some doctor was called in, who applied mustard to the feet and back of the neck, and evidently administered a mercurial, until the patient was nearly salivated. The patient still complains of the severe pains in the head and upper part of the neck. We find his head persistently thrown backward, and the patient unable to flex it; the skin cool; pulse 88, and a little fuller than natural; tongue covered with a thin white coat; and gums sore; anorexia and thirst are complained of; the bowels are said to be regular, and the urine natural. At bed-time, ordered chlor. hyd. ʒ ss.

April 2. Pain continues. The patient becomes dizzy on rising, and can hardly walk, even when supporting himself by the bed or table. Prescribed potass. brom. gr. xv, every four hours.

April 3. Herpes about the mouth; pain in the head diminishing; appetite improving; bowels regular. The patient complains of double vision when looking at objects across the ward.

April 4. Patient better.

April 6. Patient still improving. Cannabis indica continued, but no other treatment.

April 8. Patient feels very well; has very little pain, and is able to be up. Medicine discontinued.

April 9. The pain in the head is worse to-day.

April 10. Pain in head and neck increasing. Ordered tinct. cannabis ind. ʒ ss every three hours.

April 11. Pulse 104; temperature 103¼° F.; patient complaining bitterly of pain in the head and neck; head drawn backward; bowels constipated; poor appetite; considerable thirst; tongue coated yellowish white. Ordered a saline cathartic, and increased the dose of cannabis ind.

April 12. The patient felt very comfortable last evening, but the head is worse this morning. By an oversight of the nurse, the cathartic was not administered; it will be given to-day. The pain in the head is increased if the patient attempts to rise.

April 13. Pain about the same; bowels moved. Ordered potass. brom. gr. xx, every four hours, and quin. sul. gr. ii, with morph. sul. gr. 1-7, every three hours.

April 14. The patient complains less of pain in the head and neck, but complains of pain over the sacrum, which pain he says has been present for some days, though he has never before spoken of it. Discontinued the morph. and quinine, and ordered a brisk cathartic.

*Case* 6.—Wm. G——; 26 years of age; waiter; a native of Germany. Admitted February 24th.

This patient was admitted on account of a stricture of the urethra, from which he had been suffering for one year.

Eight days after admission he began to complain of thirst and loss of appetite. The pulse was somewhat accelerated, and the skin hot. Quin. sulph. gr. v, three times a day, was prescribed.

The following day (March 4th) the pulse was 120 and the respirations 23; the countenance was anxious and pinched, and the skin moist with sweating; the urine passed readily.

At 7 a. m. of the same day, the patient began to have tonic spasms; he became unconscious, and had to be restrained to prevent him from getting out of bed. He was unable to swallow, and breathed laboriously. Pulse 160, small. Two hours later the temperature in the axilla was found to be 104°, and in the rectum 107°. There was some opisthotonos; the spasms continued to recur, but more frequently when the patient was disturbed. Potass. brom. gr. xxx was administered. At this time an eruption like urticaria had appeared over most of the body. Two hours later, at eleven o'clock, the patient was more quiet, but at once went into spasms if disturbed.

March 5. The patient became quiet about two o'clock, and died at three a. m. this morning.

In this case an autopsy revealed a thickened and corrugated bladder. The pelvis of the kidneys were dilated, and a small abscess was found in the left kidney.

There was much engorgement of the sinuses of the brain, and extensive effusion of lymph into the arachnoid cavity. The lateral ventricles were nearly filled with fluid.

The spinal canal was not opened, but the membranes at the base of the brain indicated that the inflammation had extended along the spinal meninges.

Four Children at a Birth.—A woman, residing at Northampton, Mass., gave four children at a birth. The mother and children were doing well at last advices. She has had seven children within the last thirteen months.—*Med. Record.*

Conium in the Treatment of Acute Mania.—Dr. Crichton Browne has been making experiments with conium in the treatment of acute mania, and finds the result to be far more rapid and complete recovery than by any other method with which he is acquainted.

In a valuable paper in the *Lancet* Dr. Browne expresses his conviction that the conium treatment of acute mania will speedily recommend itself, to those who use it rightly, as the most efficacious mode of dealing with that form of mental disease. In order, however, to secure its benefits, two conditions must be observed; firstly, the preparation used must be good and active; secondly, the doses administered must be adequate in amount. The succus conii is certainly the most trustworthy preparation of the drug. Even the succus, however, varies in activity in an extraordinary degree. Some of it is absolutely inert. The doses must be sufficient to produce the physiological action of the drug in order to prove beneficial in disease. The effect of conium is inversely as the motor activity of the individual to whom it is given. This being so, it must be evident that in acute mania, in which motor activity is at a maximum, very large doses will be essential. Dr. Browne has given a woman laboring under acute mania as much as two ounces of succus conii at one dose, and repeated this every four hours for two days. This was, however, an extreme case, and the patient had been gradually habituated to the drug. As a rule, he has commenced with two drachms of the succus for a woman, and three drachms for a man; and rapidly increased the dose until he noticed some cessation of restlessness, or signs of lassitude or weakness of the limbs. It is rarely that a dose of one ounce or ten drachms requires to be exceeded; and sometimes improvement begins with the very first administration, in which case no increase of quantity is necessary.—*—Ibid.*

